# The Geology of Aquitards in Alluvial Aquifers: A Predictive Approach Based on Facies Models

**DOI:** 10.1111/gwat.70048

**Published:** 2026-01-26

**Authors:** Michael R. Shultz, Colin Plank

**Affiliations:** ^1^ Geosyntec Consultants, Inc. Minneapolis Minnesota; ^2^ Geosyntec Consultants, Inc. Grand Rapids Michigan; colin.plank@geosyntec.com

## Abstract

A sophisticated understanding of the three‐dimensional distribution of silt‐ and clay‐rich bodies of strata (elements) in aquifers is critical given that they not only have the potential to act as aquitards or semi‐confining units and vertically partition groundwater flow into separate aquifer zones, but also provide lateral barriers to groundwater flow, impacting contaminant distribution and groundwater flow dynamics. Additionally, when in prolonged contact with dense nonaqueous phase liquid (DNAPL) or contaminated groundwater, fine‐grained elements may become storage zones for contaminant mass via matrix diffusion and thus serve as long‐term secondary sources of contamination to groundwater that can confound remediation strategies and render remedy performance projections unreliable. The stratigraphic architecture of aquifer systems, including fine‐grained facies architecture, is complex but is not random and can be effectively predicted through application of facies models. This paper reviews depositional models (“facies models”) for common depositional environments with a focus on alluvial end‐members of braided fluvial, meandering fluvial, and alluvial fan facies models. We examine the facies models from the perspective of aquitards and present case studies to provide an overview of the expected aquitard dimensions and characteristics. The critical yet underappreciated role of the paleosol as a potential aquitard is also examined, and basic criteria for differentiating ancient floodplain clay units with high lateral continuity from other laterally discontinuous clay units are provided.

## Graphical abstract

We review facies models for alluvial aquifers and demonstrate how they can be used to predict aquifer and aquitard continuity in three dimensions. We provide case studies illustrating how facies models can be used to improve groundwater investigation and remediation outcomes.

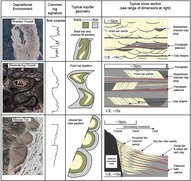

## Background

While it has long been understood that groundwater occurrence and migration is strongly controlled by the three‐dimensional stratigraphic heterogeneity (e.g., Koltermann and Gorelick 1996; Puls and Barcelona 1996; Weissmann and Fogg 1999; Shultz et al. 2017; Shultz et al. 2023), the treatment of aquifers as isotropic and homogeneous porous media persists in groundwater restoration investigations and remedy implementation. The remedial investigation (“RI”) process has focused on spatial delineation of contaminants to low concentrations and characterization of chemical composition of contaminants (commonly referred to as “nature and extent”), with little focus on understanding the details of the stratigraphy, facies architecture and associated heterogeneity. As a result, a great number of groundwater cleanups during the last half century have underperformed with respect to cleanup timeframes and other metrics (e.g., National Research Council 2013).

Meanwhile, having recognized the financial risks to resource production and major capital project economic success posed by geologic heterogeneity, the oil and gas industry was developing predictive approaches to assess continuity and dimensions of reservoir and non‐reservoir facies through the application of sequence stratigraphy and facies models (e.g., Van Wagoner et al. 1990, Walker and James 1992, Posamentier and Walker 2006, Feldman 2024). These concepts are now being increasingly employed at a variety of scales to address groundwater problems and particularly per‐ and polyfluoroalkyl substances (PFAS) contamination of aquifers (e.g., Ehman and Cramer 1997; Shultz et al. 2017; Shultz et al. 2023; Sadeque and Samuels 2024). This geologically focused approach to groundwater remediation has the potential to improve the efficiency of cleanup efforts for PFAS relative to historical chlorinated solvent cleanup efforts. Furthermore, in nations where groundwater remediation is at an earlier phase compared to the United States, cleanup efforts in those nations may be streamlined by avoiding reliance on assumptions of homogeneous, isotropic aquifer conditions.

## Hydrostratigraphic Units

The term “hydrostratigraphic unit” has been used in various contexts by various authors, from scales commensurate with regional geologic formations (e.g., the St. Peter Sandstone, sensu Maxey 1964), to the scale of individual aquifer compartments present at a particular groundwater restoration project site (e.g., Noyes et al. 2001). For the purposes of this review, we consider a hydrostratigraphic unit to refer to a body of water‐bearing strata that has a high degree of internal hydraulic interconnectivity and is bounded laterally and vertically by relatively impermeable units. The relatively impermeable units significantly limit lateral and vertical hydraulic communication, and the shape and dimensions of the hydrostratigraphic unit exert control over groundwater flow vectors and contaminant migration (e.g., Shultz et al. 2023).

## Overview of Depositional Environments and the Origin of Aquitards

**Figure 1:**
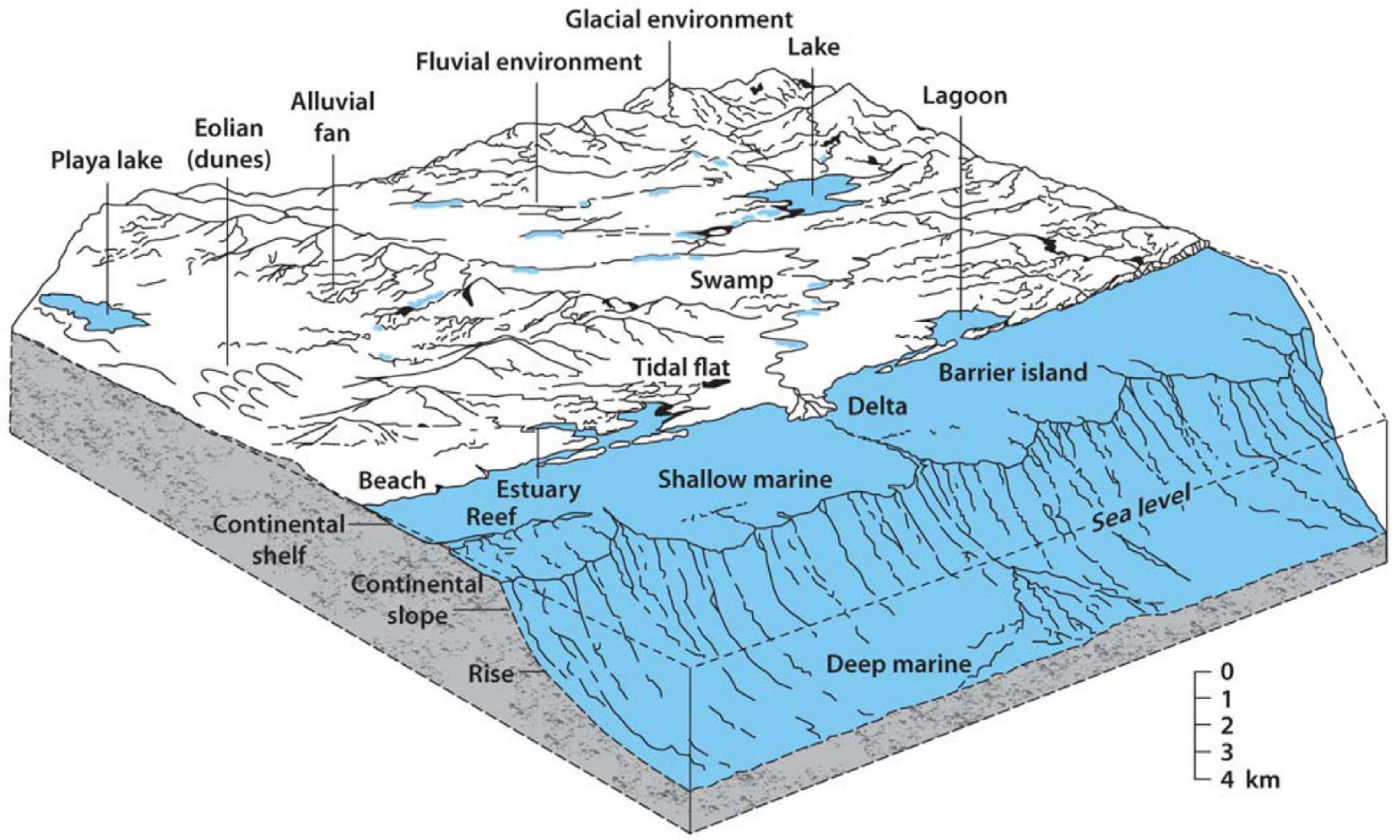
Block diagram depicting most common depositional environments from alpine glaciers to open marine (From Shultz et al. 2017).

Depositional environments refer to geographic areas where sediments are transported, deposited, buried, and preserved by natural sedimentary processes (Figure 1).

Due to the processes acting to transport and distribute the sediments, each environment has characteristic building blocks, or “architectural elements”, which stack together in predictable patterns. When buried by younger sediments and saturated with groundwater, these deposits form aquifers and aquitards in the subsurface. Table 1 presents the basic log motifs, basic architectural elements (aquifer and aquitard), general dimensions, impact on the conceptual site model, and data resolution needs corresponding to a variety of the most common depositional environments.

**Table 1:** Most common depositional environments and associated aquifer and aquitard characteristics and dimensions (modified from Shultz et al. 2017). Note that in defining general body dimensions in X, Y, and Z coordinate space, the X dimension is generally regarded as perpendicular to the direction of the primary force moving sediment (e.g. stream flow, wave energy), the Y dimension is parallel to that force, and Z is vertical.

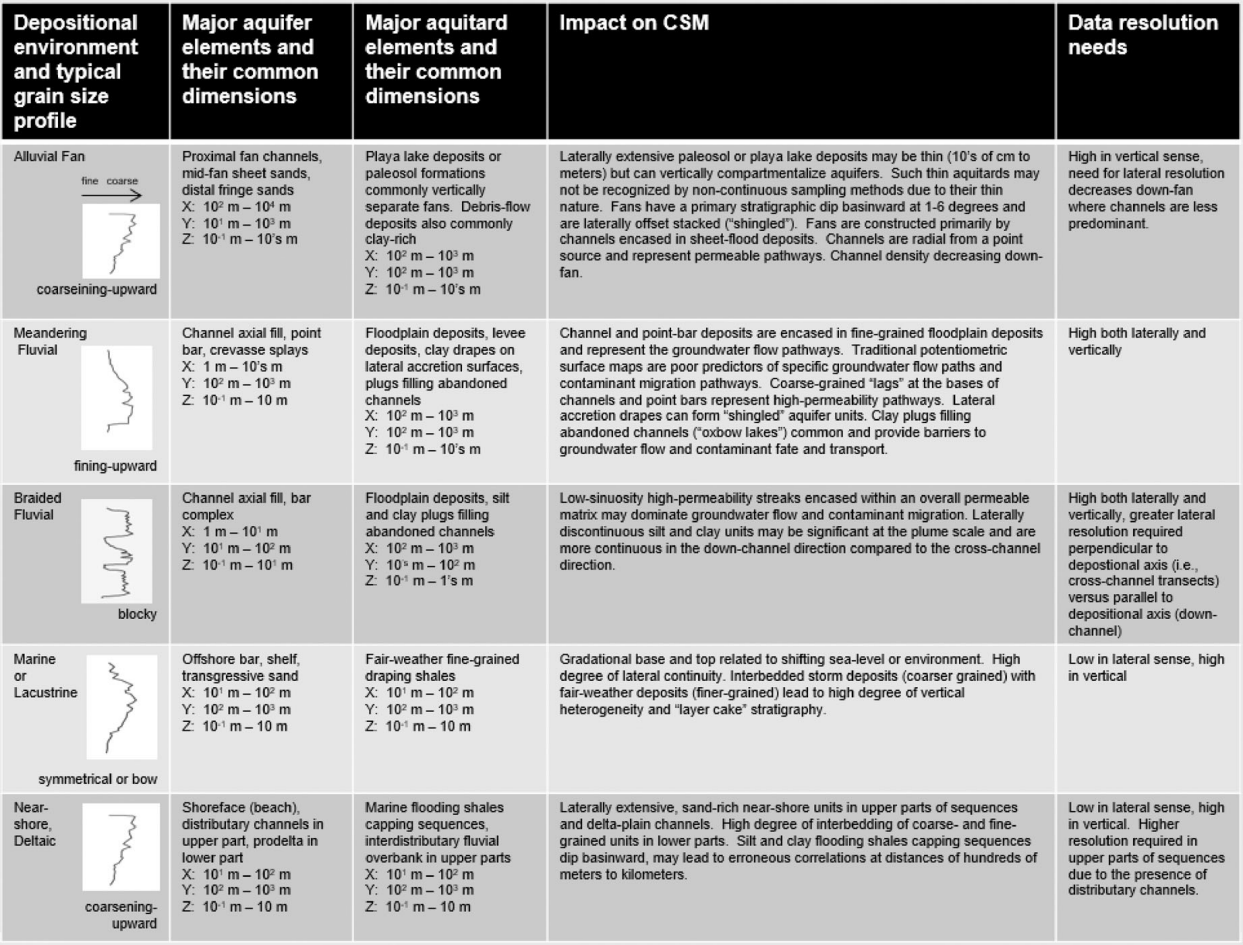

**Figure 2:**
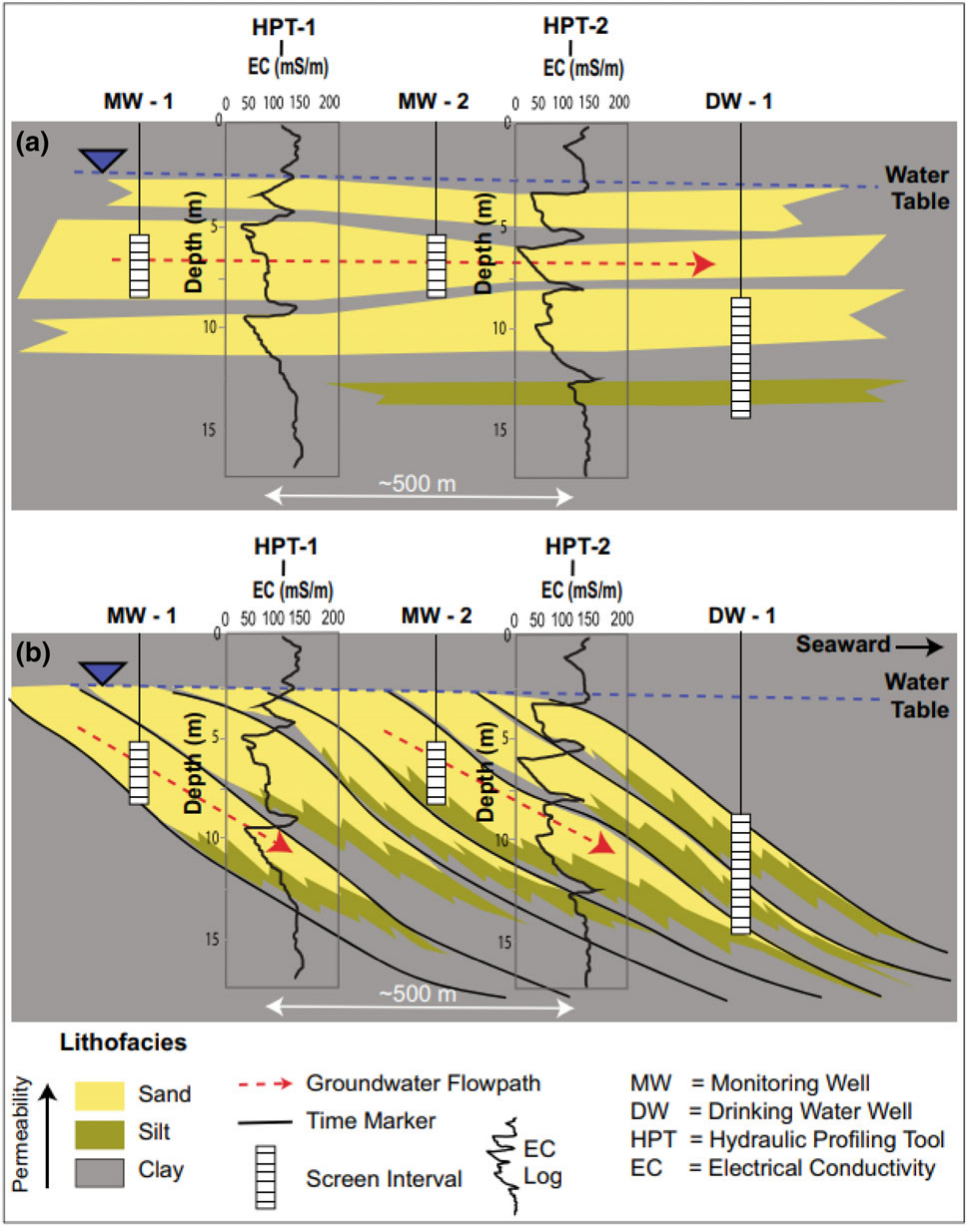
(a, b) Comparison of an incorrect “layer‐cake” lithostratigraphic correlation of sand units (top) versus a correct correlation of sand units based on knowledge of deltaic facies models which predict seaward‐dipping correlations (From Sadeque and Samuels 2024). Correlation in (a) would be appropriate in a paleoshoreline‐parallel orientation, but not in a paleoshoreline‐perpendicular orientation.

Knowledge of the organization of architectural elements provides powerful predictive capabilities for assessing subsurface correlation and aquifer and aquitard arrangement. Figure 2 presents two alternative correlations (Figure 2a and 2b) of two electrical conductivity logs acquired from aquifers deposited in a fluvio‐deltaic depositional environment. The logged wells are located ~500 ft apart, in the direction of depositional dip. The figure demonstrates how a “lithostratigraphic” layer‐cake correlation of sand and clay units is fundamentally flawed in a deltaic depositional environment. It is well known that in such a deltaic setting sand bodies typically show coarsening‐upward profiles, and dip downward in the direction of delta outbuilding (i.e., the direction of “progradation”). This example illustrates the need for expertise in stratigraphic sciences and an understanding of depositional environments to make geologically realistic correlations between wells.

Facies models have been developed over the last half century or so in very detailed fashion for a wide range of depositional environments, and the reader is referred to the scientific literature for further reading (e.g. Feldman 2024). It is beyond the scope of a single review paper to address dimensional aspects of aquitards in the full range of depositional environments depicted in Figure 1. Facies models generally are focused on “end member” examples of environments, and here we focus on three end‐members of alluvial environments, namely braided fluvial, meandering fluvial, and arid alluvial fan. However, it is important to recognize that there exists a continuum of styles dependent on many factors and all sites are somewhat unique. Additionally, due to changing conditions through time, a site may contain aquifers and aquitards corresponding to more than one depositional environment. For instance, many of the modern meandering stream systems in river valleys of the Midwestern region of the United States are underlain by braided stream deposits laid down during times of higher river discharge at the end of the Last Glacial Maximum (e.g., Anderson et al. 2021).

## Alluvial Settings

Alluvial depositional environments refer to terrestrial environments in which sediments are transported and deposited primarily by flowing water (perennial and ephemeral rivers and streams) and debris flow. Table 2 presents general information regarding morphology, architectural elements and common dimensions of aquitard and aquifer elements corresponding to braided fluvial, meandering fluvial, and alluvial fan depositional environments.

**Table 2:** Common log signatures, aquifer distribution, cross sections, and dimensions of aquitard elements according to depositional system type.

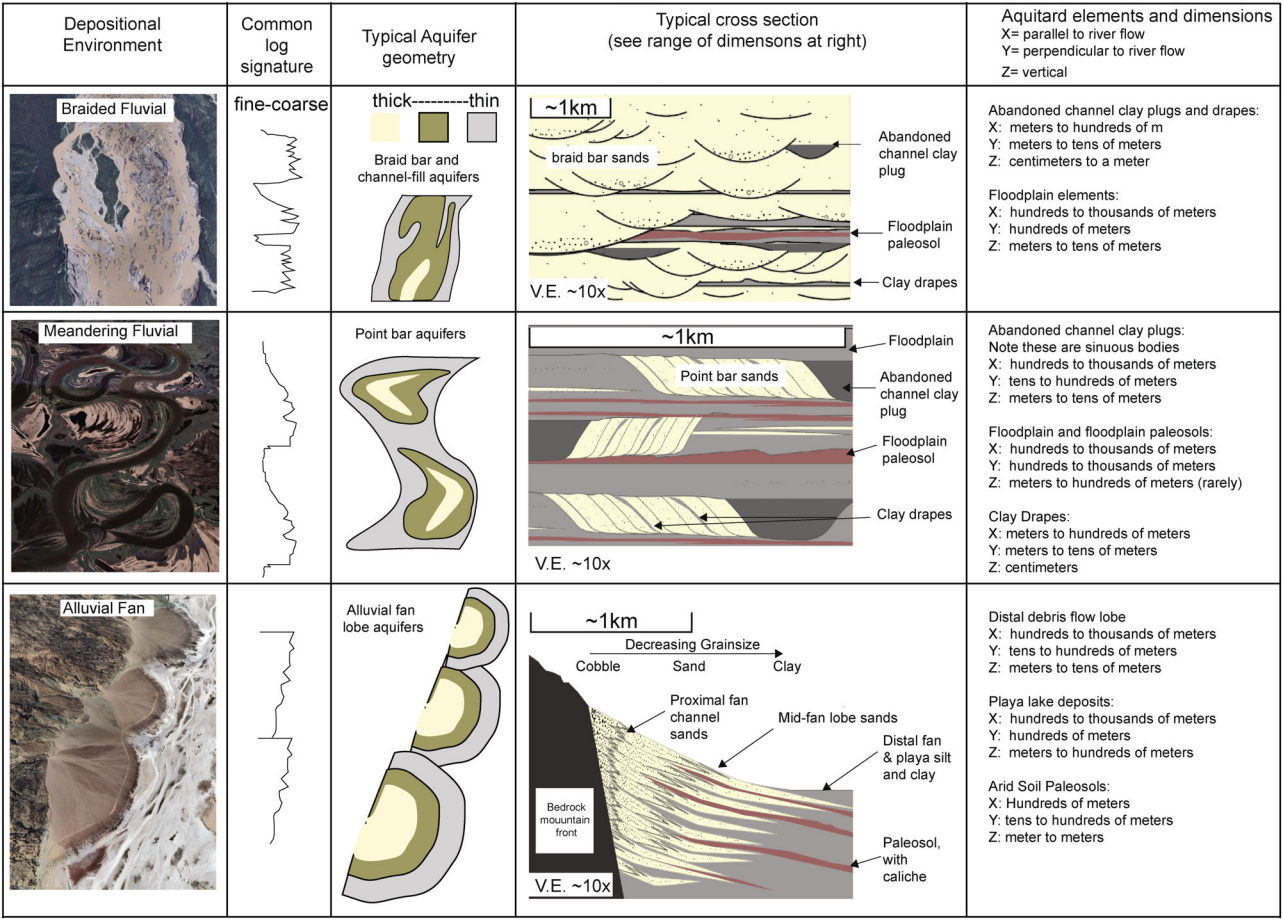

A great many contaminated groundwater sites are situated within major river valleys due to the presence of abundant freshwater resources required for industrial processes and proximity to transportation infrastructure. As such, fluvial facies models are exceedingly important tools for predicting aquifer architecture in these areas. Fluvial systems can be broadly subdivided into two end‐members of meandering and braided morphology, each discussed below. However, in reality, a continuum of styles exists between meandering and fluvial end‐members (e.g., Holbrook and Allen 2020).

In mountainous and arid regions worldwide, alluvial fans form important aquifers. Note that alluvial fans are also an end‐member of a continuum of environments referred to as “distributive fluvial systems” (e.g., Hartley et al. 2010), which comprise combinations of alluvial fans and other fluvial systems (typically braided‐type systems).

## Paleosols in Alluvial Systems

There exists a common misperception in the groundwater community that paleosols are only relevant to studies of regionally correlative and well‐documented markers of major climatic events such as interglacial epochs (e.g., the Sangamon paleosol which is a widespread marker horizon throughout much of the Central United States). However, in addition to the well‐known regionally correlative paleosols, there are a great many locally correlative paleosols present within any given alluvial deposit. Furthermore, it is not uncommon for paleosols to form a significant proportion of the overall stratigraphic section of an alluvial deposit (e.g., Bown and Kraus 1987). These paleosols represent soils that developed during periods of non‐deposition or slow deposition when active deposition was occurring elsewhere in the basin. Following soil development (pedogenesis) in one area, active, rapid deposition may resume in that location due to the dynamics of the depositional environment (e.g., due to channel avulsion or abandonment), unrelated to climate shifts (e.g., Wright et al. 2024). Such processes are referred to as “autocyclic” processes, as they are related to the natural dynamics of a specific depositional system, as opposed to “allocyclic” processes which result from extra‐basinal forcing factors such as sea‐level change, tectonic uplift events, or climate change (e.g., Cecil et al. 2004). Many paleosols formed by autocyclic processes, while not regionally correlative, may be significant with respect to groundwater flow (e.g., Weissmann and Fogg 1999) and contaminant fate and transport both from the perspective of their potential function as a hydraulic barrier and their potential utility to a stratigrapher as a chronostratigraphic surface or marker bed (Catuneanu 2006).

Recognizing paleosol horizons when performing environmental investigations can be a challenge, especially given the fact that recovery of continuous undisturbed core is sometimes not possible or may not be part of the work plan due to increased cost. The general ongoing failure to recognize and log paleosols is perhaps exacerbated by lack of training in their recognition via color, horizonation, and pedogenic structure (e.g. Cleveland and Driese 2024). Staff commonly tasked with boring logging are not commonly trained to identify paleosols, and they are often trained practically that everything they are logging is “soils,” which is in actuality almost never the case. In essence, you find only what you are looking for when drilling, and the environmental industry is not currently adept at this aspect of stratigraphic characterization.

## Braided Fluvial Systems

In fluvial systems characterized by an abundance of sediment supply and highly variable streamflow rates, the surface water flow velocity is great and braided type systems develop. Braided streams are also commonly found in topographically confined river valleys. Braided streams have wide, shallow channels which branch and rejoin, separated by mid‐channel bars that migrate during high‐flow periods (Table 2). Braided river deposits comprise prolific aquifers worldwide due to the abundance of coarse‐grained sand and gravel with few lateral barriers to groundwater flow (e.g., parts of the Ogallala Aquifer). However, outside of the main active braid plain, extensive floodplains may be present. These floodplains are composed of fine‐grained material and, when preserved and buried by younger braided streams, may form extensive aquitards. Aquitard facies present within braided river systems include not only floodplain deposits but also include minor silty mud plugs confined within abandoned channels, clay drapes deposited during slack‐water conditions, and interbedded silts and clays on braid bar tops.

## Meandering Fluvial Systems

**Figure 3:**
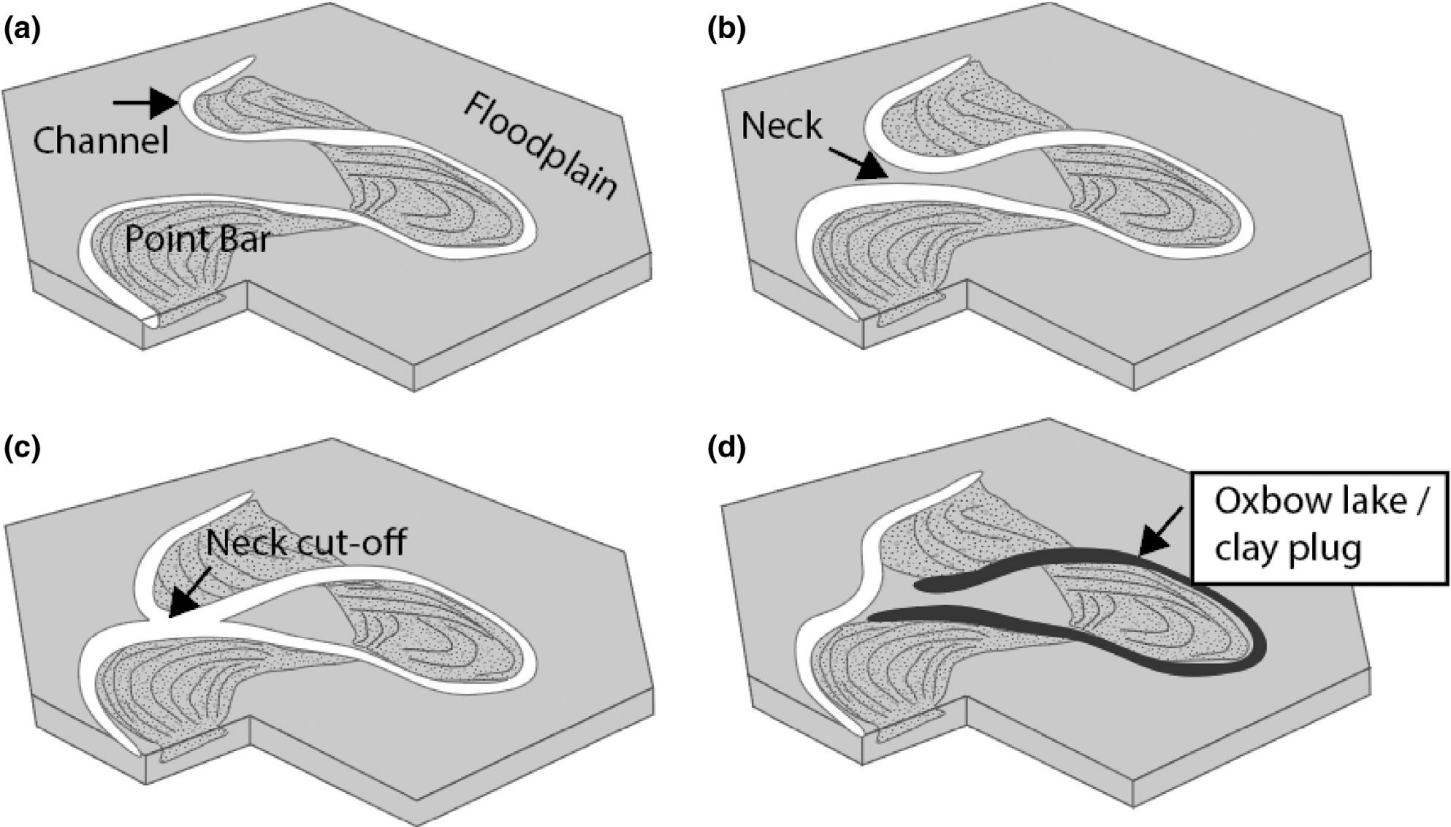
Time series block diagrams of meandering river evolution illustrating the two main clay‐prone elements of meandering streams. (a) As a result of the higher current velocity on the outer bend (“cutbank”), the river is erosive and gradually erodes the older floodplain deposits and migrates toward the outer bend. As this migration occurs, a “point bar” is deposited. (b) As the two meander bends encroach upon one another, a “neck” is formed. (c) once the two meander bends meet, a “neck cut‐off” occurs, river flow is diverted, and the meander bend is abandoned as an “oxbow lake.” (d) oxbow lakes are slowly filled with fine‐grained sediment following formation.

In fluvial systems characterized by lack of topographic confinement, low gradient and the presence of a wide variety of grain sizes from pebbles to clay (“mixed‐load” system), the river migrates laterally (“meanders”) across the landscape producing point bar deposits (Figure 3).

When one channel cut bank erodes to the point where it encounters the channel associated with another meander bend, the upstream bend of the river is “cut off,” resulting in an abandoned, curved stretch of river known as an “Oxbow Lake” (Figure 3). Over time, and once isolated from the main river axial channel, oxbow lakes become filled with fine‐grained sediments such as silt and clay, referred to as “clay plugs.” Clay plugs are well‐known to form competent barriers to lateral fluid flow in the subsurface. This is demonstrated by distinct oil/water contacts and pressure regimes in oil and gas fields (e.g., the Little Creek/Sweetwater oil and gas field in Mississippi [Werren et al. 1990]), and by deflection of contaminant plumes in groundwater (Shultz and Champion 2015).

**Figure 4:**
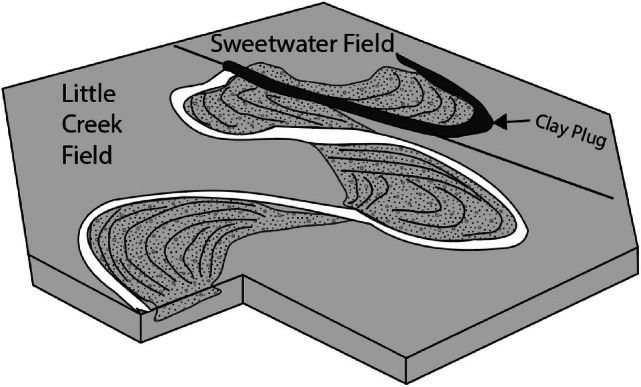
The Little Creek Field consists of several hydraulically connected point bar deposits, while the Sweetwater Field is isolated from the Little Creek Field by a clay plug (oxbow lake deposit). The hydraulic isolation of the fields from one another is demonstrated by different oil water contacts and pressure data (Werren et al. 1990).

The Little Creek/Sweetwater oil and gas field and in provides a powerful demonstration of the capacity of the oxbow lake deposit (“clay plug”) to form a lateral barrier to fluid flow, and the capacity for the floodplain deposits to form a vertical flow is well‐demonstrated as the top seal to prevent upward migration of oil and gas to the surface. Key aquitard types which are abundant in meandering fluvial systems: the floodplain aquitard and the oxbow lake clay plug aquitard (Figure 4).

Note that the reservoir sands are completely encased in floodplain clays on the top, base, and laterally. The aquitard dimensions are therefore hundreds of square kilometers laterally and tens to hundreds of meters vertically. In addition to the floodplain aquitard demonstrated here, the oxbow lake clay plug aquitard represents an important lateral barrier to flow, as the smaller Sweetwater field is isolated as evidenced by hydrocarbon production and pressure data (Werren et al. 1990).

## Fluvial System Case Study: The Fallacy of the “A Aquitard”

**Figure 5:**
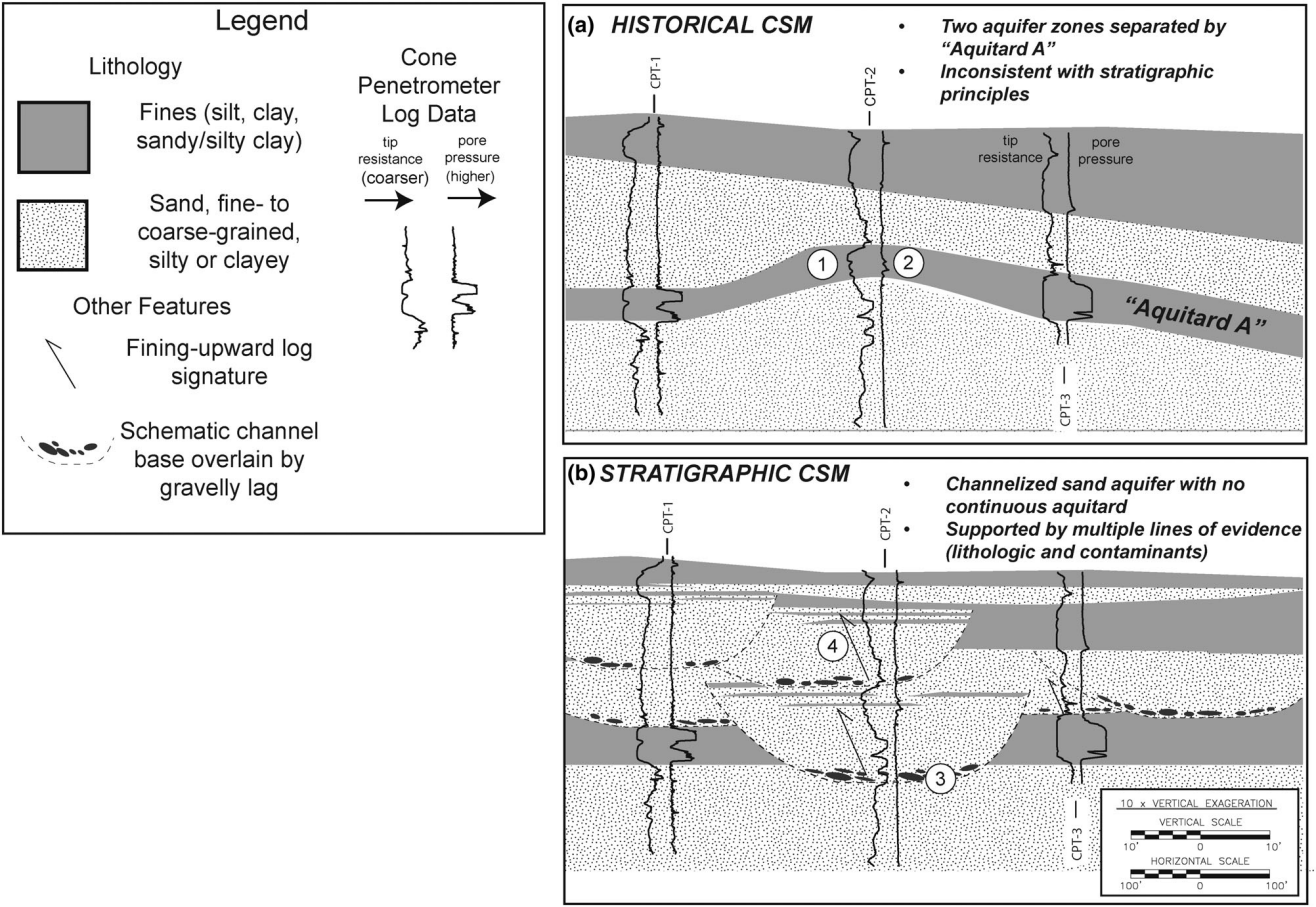
Comparison of two interpretations of three cone penetrometer logs. At a site in the northeastern United States, the historical CSM had interpreted two aquifer zones separated by a continuous aquitard referred to as “Aquitard A” (Figure 5a). This interpretation formed the CSM that had been used as a basis for monitoring and remedy implementation for decades. However, this interpretation is inconsistent with stratigraphic principles. A more realistic stratigraphic interpretation is that of a channelized sand aquifer which has completely eroded the clay unit in the vicinity of CPT‐2 (Figure 5b). The stratigraphic CSM is supported not only by stratigraphic principles but also by the following (numbered circles on cross sections): (1) Positive relief (mounding) of a clay unit is not a common stratigraphic geometry and calls the interpretation into question. (2) Pore pressure response on CPT minimal compared to high pressure response in CPT‐1 and CPT‐3, suggestive of thin silt beds within the upper part of a fining‐upward channel‐fill sequence, supported by boring log descriptions from co‐located monitoring well. (3) Presence of gravel‐bearing zone at the base of sand is indicative of a basal channel “lag.” (4) Fining‐upward log profiles characteristic of channel‐fill deposits.

An outgrowth of the historical engineering approach to subsurface investigations for aquifer assessment and remediation is the perpetuation of a layer‐cake conceptual site model and often the perception of a continuous aquitard that is protective of deeper aquifer units (referred to herein as the “A Aquitard”). Often, the aquitard does not exist as a continuous barrier, and the perpetuation of the fictitious “A Aquitard” results from a combination of poorly resolved field data collection or correlation of discontinuous fine‐grained lenses as one continuous layer. Figure 5 illustrates such a case where the existence of an aquitard was a decades‐long mainstay of the Conceptual Site Model (CSM). The existence of the aquitard was so firmly ingrained in the site history that it formed the basis for subdividing the aquifer and mapping contaminant plumes in different aquifer zones corresponding to different elevations.

However, when site cross sections were reviewed through the lens of facies models, the interpretations of the continuous “Aquitard A” violate a stratigraphic “rule of thumb” that correlative clays tend not to have a “mounded” shape, and this calls the correlation into question (Shultz et al. 2017). Upon detailed examination of Cone Penetrometer Testing (CPT) data collected in the area, it is apparent that the fine‐grained unit interpreted as Aquitard A is discontinuous and has been completely removed by an erosive channel at CPT‐2, and the correlation of the A Aquitard was incorrect, and the A Aquitard is in fact discontinuous at the site.

## Alluvial Fan Environments

**Figure 6:**
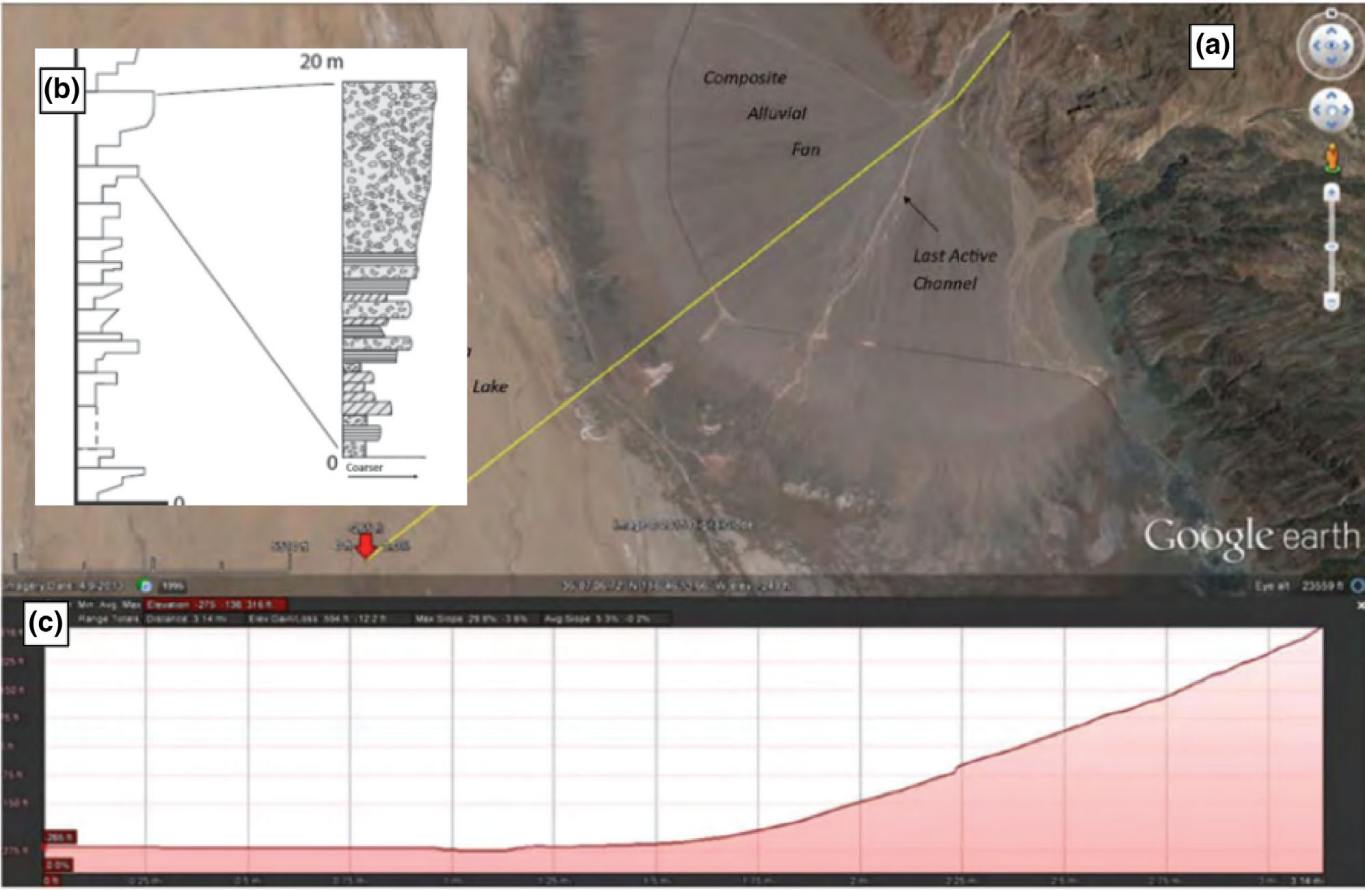
(a) Google Earth image of an alluvial fan in Death Valley, California, USA. (b) As the fan builds out, it leaves cyclic coarsening‐upward sequences of sand and gravel stacked one atop the other. (c) Topographic profile of the fan illustrating slope of fan in the direction of sediment transport.

Alluvial fans form along mountain fronts where high‐energy, flashy streams debouche onto the basin floor. Where this occurs, streamflow energy decreases very rapidly and the coarsest sediment (boulders, gravels) are deposited at the head of the fan and progressively finer materials are carried further down‐fan (Figure 6). For a detailed review of alluvial fans and associated facies models, the reader is referred to textbooks and scientific literature (e.g. Blair and McPherson 1994; Feldman 2024).

In the distal end of the fan, known as the “toe,” the fan interfingers with fine‐grained silt and clay known as the “playa.” As the areas where sediment is actively being deposited (“lobes”) switch locations, individual coarsening‐upward cycles are stacked upon one another. When an active lobe is abandoned, a soil may form separating individual lobes. After burial, these soil horizons may be preserved as “paleosols,” which have the potential to act as aquitards (e.g., Weissmann and Fogg 1999). This may act in conjunction with rising water levels in playa lakes, further enhancing the aquitard potential of the paleosols.

## Alluvial Fan Case Study: Paleosols as Aquitards

**Figure 7:**
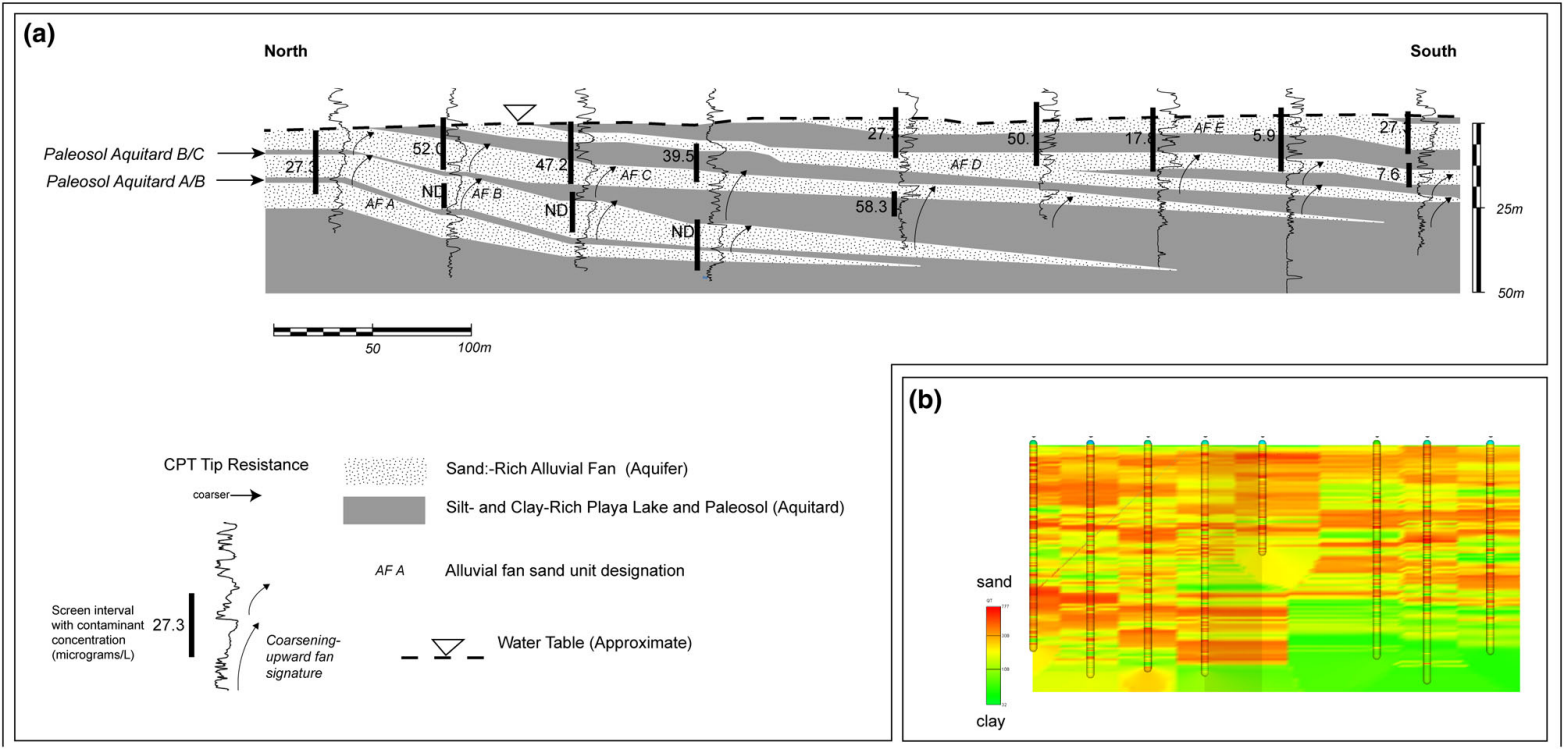
(a) Stratigraphic cross section illustrating stacked alluvial fans. (b) Same data as (a) kriged via a computer algorithm. Refer to text for discussion.

At a groundwater remediation site in the desert southwest of the United States, a series of alluvial fans represent groundwater aquifers. Figure 7a shows a geologic cross section prepared using a combination of cone penetrometer data, boring log lithologic data, and geochemical data from groundwater samples. The section illustrates stacked, coarsening‐upward profiles of a series of alluvial fans interfingered with playa lake deposits. The alluvial fans advanced outward in numerous pulses and then were inundated rapidly, likely due to Pleistocene glacial cycles and rapid growth of glacial lakes. In the northern end, the “proximal” end, these clay units are thin, likely representing paleosols separating individual fan deposits. Where wells are screened within individual alluvial fans, distinct contaminant concentrations are observed, demonstrating the potential of paleosols of limited thickness (<50 cm) to restrict groundwater flow and compartmentalize aquifers. Figure 7b illustrates the same CPT data correlated with a computer via a kriging algorithm. Due to the dipping nature of the hydrostratigraphic units, it is apparent that the kriged section misrepresents the aquifer architecture and specifically the long correlation length of the thin clay beds. The kriged data section was used as the basis for design of an in‐situ injection remedy performed prior to the stratigraphic interpretation. The remedial action, ethanol injection, inadvertently produced a byproduct plume via injection into unimpacted groundwater in alluvial fan unit B, necessitating further remedial action. This case study illustrates the necessity of a facies‐based interpretation of lithologic data for successful remedy planning and execution.

## Practical Field Application: Clay Encountered when Drilling

When drilling for groundwater resource evaluation or groundwater contamination studies, and an unexpected clay unit (potential aquitard) is encountered it often poses a critical decision point for the project team. Project teams often struggle with the decision to continue drilling due to the risk of creating a vertical pathway through the potential aquitard for contaminants to migrate to deeper aquifer units. Teams may adjust drilling and casing plans to prevent cross‐contamination, which may increase project complexity, time, and cost. Knowledge of depositional environments provides a predictive tool to assess the likelihood of the encountered clay representing a significant, widespread aquitard.

For example, during a remedial investigation for PFAS in a sandy alluvial aquifer, encountering a fine‐grained unit requires careful interpretation. The stratigraphic features of the clay can help distinguish between a localized abandoned channel clay drape or plug—unlikely to act as a significant aquitard—and a floodplain deposit, which may be laterally extensive and protective of underlying aquifers. Since floodplain deposits often exhibit signs of soil formation, identifying pedogenic features becomes a key tool in differentiating them from clay plugs. This distinction enables the use of facies models to predict subsurface conditions beyond the wellbore. Table 3 is presented with some criteria which may be used to differentiate floodplain deposits with high potential for extensive lateral continuity versus clay plug deposits associated with abandoned channels which have limited lateral extent but potential to form strong barriers to lateral flow of groundwater and divert contaminant migration direction from the regional groundwater flow direction.

**Table 3:** Criteria for Differentiating Fine‐Grained Units Based on Observation of Core Data.

Fine‐Grained Unit	Floodplain Deposit	Abandoned Channel Clay Plug
Lateral extent and hydrogeologic implication	Potential widespread local or regional aquitard or semi‐confining layer	Limited aerial extent, possible lateral barrier to groundwater flow
Texture	Clay with silt and minor fine‐grained sand	Clay with silt, laminated
Color	Multicolored, variegated with reds, green, brown, gray, black, yellow	Dark gray to black, blue or dark yellow
Paleosol development	Evidence of paleosol formation throughout Multicolored Presence of concretions/nodules Silurian and older rocks are not rooted, bioturbated, or show signs of soil formation (pre land plants)	Little evidence for paleosol development, or paleosol development only at the top of the unit
Fossils and bioturbation	Terrestrial fossils Root casts or root remnants mammalian or other terrestrial burrows	Aquatic (fish scales common) well‐preserved plant debris and other organic matter

## Conclusions and Future Directions

While complex, aquifer architecture including fine‐grained unit distribution is not random. Facies models provide a means to predict the types of fine‐grained units present in any given site, as well as the likelihood of those units forming significant aquitards. Understanding the depositional environment of an aquifer may be accomplished through regional studies or may be accomplished through the identification of stratigraphic features and trends in vertical grain size profiles.

The geologic concepts reviewed herein also provide important constraints on numerical models of aquifers developed for simulation of groundwater flow and contaminant plume migration. Ongoing work is focused on developing advances in numerical modeling of groundwater flow and contaminant fate and transport (Einarson 2025). These authors stress the importance of capturing key elements of geologic detail at the foundational level in numerical modeling. Conventional finite difference and finite element groundwater model software such as MODFLOW models (utilizing rectangular grids) are longstanding tools in the industry. Numerical models generally require grids or cell meshes to define stratigraphic heterogeneities geometrically and via parameterization. For example, model parameters such as hydraulic conductivity in X, Y, and Z directions are defined for grid cells, forming the foundation for computations. Advanced discretization methods, such as unstructured grids, are being incorporated into popular groundwater modeling software packages, allowing numerical models to more accurately incorporate the complex geologic and hydrogeologic conditions and concepts such as we describe here. The need for synergy between hydrogeologists (e.g., their ability to recognize, interpret and communicate the reality of the subsurface) and modelers (with advancements in their ability to incorporate geologic complexity) will increase in importance in the future.

It has become apparent that high‐resolution data are required for adequate site characterization, and acquisition of high‐quality, high‐resolution data early in a project lifecycle (e.g., collection of highly resolved, data‐rich “golden spike” wells) is critical to efficient characterization and remediation of groundwater sites (e.g., Cramer et al. 2025). Having the necessary data up front, as may be provided with a “golden spike” CSM well, can allow for accurate identification of depositional environments, calibrate other proxy data such as geophysical logs, clarify the vertical and lateral data resolution needs for subsequent wells, and therefore frame the expectations for the existence and role of aquitards and preferential pathways in the aquifer (e.g., Shultz et al. 2017).

Advances in direct‐push technology for high‐resolution data collection have enhanced the ability to detect vertical stacking patterns of grain size in the subsurface and thereby more confidently identify depositional environments. These tools are increasingly employed to collect transects of logs perpendicular to and parallel to groundwater flow paths, providing superior data sets for stratigraphic interpretation where depth of investigation is relatively shallow. Geophysical logging of existing monitoring wells through casing (e.g., natural gamma ray, conductivity, or nuclear magnetic resonance logging) can also provide high‐resolution vertical data where boring log descriptions are poorly resolved (e.g., discrete depth samples instead of continuous coring). Geophysical logging of existing monitoring well networks provides a cost‐effective means of acquiring extensive suites of high‐resolution data to dramatically improve interpretations of three‐dimensional hydrostratigraphy at sites. In addition to downhole geophysical methods, surface‐based geophysical methods are becoming increasingly employed for remediation assessments. While surface‐based resistivity profiling has historically been viewed unfavorably by some, we feel that this is likely a result of the technique having been oversold in some cases, with the expectations mismatched to the technology's limitations. However, it is clear that in the right settings, such data can be very enlightening, especially when calibrated to subsurface well data and interpreted in the context of the depositional environments.

Additionally, there is a renewed focus on the quality of description of cores recovered during drilling, and an emphasis on the collection of stratigraphically useful data for the identification of depositional environments (Meyer et al. 2022), which represents a significant advancement in data collection methodology in the groundwater community. Moving forward, a greater focus on the identification of depositional environments and the application of facies models to groundwater sites has the potential to improve project outcomes, efficiency, and protectiveness of human health and the environment worldwide.

## Data Availability

Data sharing not applicable to this article as no datasets were generated or analysed during the current study.
